# Optogenetic regulation of transcription

**DOI:** 10.1186/s12868-018-0411-6

**Published:** 2018-04-19

**Authors:** Oksana Polesskaya, Ancha Baranova, Sarah Bui, Nikolai Kondratev, Evgeniya Kananykhina, Olga Nazarenko, Tatyana Shapiro, Frances Barg Nardia, Vladimir Kornienko, Vikas Chandhoke, Istvan Stadler, Raymond Lanzafame, Max Myakishev-Rempel

**Affiliations:** 1 University of California, San Diego, CA USA; 2 Research Center for Medical Genetics RAMS, Moscow, Russia; 3 Center for the Study of Chronic Metabolic and Rare Diseases, School of Systems Biology, George Mason University, Fairfax, VA USA; 4 Localized Therapeutics, LLC, San Diego, CA USA; 5 Rochester General Hospital, Rochester, NY USA; 6 Raymond J. Lanzafame, MD PLLC, Rochester, NY USA; 7 Vaccine Research Institute of San Diego, San Diego, CA USA; 8 Atlas Biomed Group, Moscow, Russia; 9 Moscow Institute of Physics and Technology, Dolgoprudny, Russia

## Abstract

Optogenetics has become widely recognized for its success in real-time control of brain neurons by utilizing non-mammalian photosensitive proteins to open or close membrane channels. Here we review a less well known type of optogenetic constructs that employs photosensitive proteins to transduce the signal to regulate gene transcription, and its possible use in medicine. One of the problems with existing gene therapies is that they could remain active indefinitely while not allowing regulated transgene production on demand. Optogenetic regulation of transcription (ORT) could potentially be used to regulate the production of a biological drug in situ, by repeatedly applying light to the tissue, and inducing expression of therapeutic transgenes when needed. Red and near infrared wavelengths, which are capable of penetration into tissues, have potential for therapeutic applications. Existing ORT systems are reviewed herein with these considerations in mind.

## Background

Chemical regulation of transcription is widely used in research settings, e.g., the tetracycline-regulated transcription system [1] is a popular tool for animal based functional studies. The typical workflow of chemically regulated transcription requires construction of externally regulated promoters that are then inserted into cultured cells or animal tissues. When the modified cells are treated with a chemical inducer, the engineered promoter is either stimulated or repressed.

Optogenetics initially emerged as a tool for light-inducible activation or suppression of membrane channel currents in the neurons of various subtypes. By combining light-sensing module and transcription factor module optogenetic tools can be used to regulate gene transcription. The first study describing optogenetic regulation of transcription (ORT) was published in 2002. Shimizu-Sato et al. [2, 3] employed red light-induced binding of the plant photoreceptor phytochrome to the phytochrome interacting factor 3 (PIF3) and the reversal of this binding by far-red light to induce GAL4 promoters in yeast. More recently, ORT systems were demonstrated in both neurons and non-neuronal cells and tissues of mammals [4–6]. Initially, optogenetic systems aimed to fill a niche as the method of choice for studying the pathophysiology of neurological and neuropsychiatric disorders in a range of animal models [7]. In the course of the last decade, optogenetics gained much popularity in research not limited to the field of neurobiology [8, 9]. Moreover, the current renaissance in gene therapy calls upon optogenetic approaches for patients with medical conditions that lack appropriate treatments, such as pharmacoresistant epilepsy [10] and inherited retinal degeneration [11]. High tissue penetration of the red and NIR light makes optogenetic approaches attractive for tissues where NIR light can be efficiently delivered by light emitting diodes (LED) [12, 13].

### Progress of gene therapy

Use of ORT in therapy would require gene delivery into the affected site in the body. The acceptance and approval of gene therapy has been slow, but in recent years clinical trials show promise in several diseases, especially cancer. A notable example is Chimeric Antigen Receptor (CAR) T Cell Therapy [14], which uses genetically modified cells to express a transgene and attack specific cancer cells. Two CAR-based therapies, CTL019 by Novartis and JCAR01 by Juno, were granted “breakthrough status” in 2014 [15].

The first approval of gene therapy took place in China in 2003, when an oncolytic recombinant adenovirus engineered to express wild type p53 (Adp53), also called Gendicine, was introduced to treat head and neck squamous cell carcinoma [16]. The adeno-associated virus-based Glybera [17] was approved for the therapy of rare familial lipoprotein lipase deficiency in 2012 in Europe, and is currently undergoing clinical tests for possible approval in the US. In 2016, a second gene therapy treatment, Strimvelis, was approved in Europe for adenosine deaminase deficiency, a form of severe combined immunodeficiency [18]. LentiGlobin BB305, a gene therapy for β-thalassemia, was granted “breakthrough status” in 2015 [19]. This therapy inserts a gene construct into hematopoietic stem cells (HSC) ex vivo, followed by reintroduction of the resulting engineered HSC cells to the patient. Optogenetic-based therapy for treating retinitis pigmentosa is currently undergoing clinical trial (ClinicalTrials.gov Identifier: NCT02556736). This treatment is based on expressing channelrhodopsin-2 (ChR2) in retinal neuronal cells other than rods, turning them functionally into artificial photoreceptors. The growing acceptance of gene therapy has provided the incentive to examine a number of novel approaches, such as light-driven expression of therapeutic proteins.

### Choice of wavelength

**Fig. 1:**
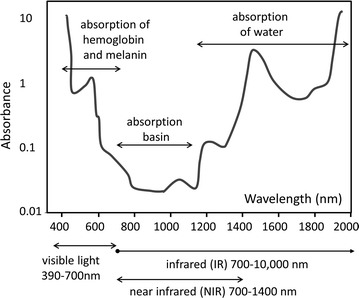
Tissue penetration spectrum. The range where light penetrates deepest in the tissue, also known as the absorption basin as the phototherapeutic window is located in the range of 750–1100 nm in the near infrared (NIR) part of the spectrum

The choice of promoter-activating wavelength is dictated by two parameters: safety and tissue penetration. The visible light spectrum ranges from 400 to 750 nm. Shorter wavelengths (UV light) do not penetrate more than a millimeter and are harmful for cells [20]. Red (620–750 nm) and a part of the near infrared light range (750–1100 nm) have the deepest tissue penetration [21, 22]. Figure 1 shows the “absorption basin” that represents the so-called therapeutic window with the highest tissue penetration. Only certain wavelengths are suitable for future therapeutic use, depending on the location of the target tissue. Blue light, which penetrates into the tissues to the depth of about 1 mm, is the most efficient in cell culture, tissue explants and the surface of the skin. On the other hand, red/near infrared light is capable of reaching tissues at much deeper levels and is the most suitable for therapeutic applications.

### Light detectors

Non-mammalian light-sensing proteins are used to detect light and activate or repress transcription of a transgene. Protein sensors are genetically incorporated into the mammalian cell, where they act as light-controlled switches for gene expression. Induction of transcription by light has advantages over chemically driven transgene activation due to its non-invasive nature and potential for greater spatio-temporal control [7, 23]. Light-controlled gene expression cassettes have been developed for bacterial, yeast, and mammalian cells [24, 25], making optogenetic tools valuable research instruments. Importantly, optogenetic approaches seem suitable for biomedical interventions, as they bypass the use of chemical inducers and could manipulate transcription rates of therapeutic transgenes to regulate biosynthesis of a therapeutic protein in the body. In this review, we will group these systems according to their activation light wavelengths, since the wavelength is critical for the depth of penetration of light into the body.

## Blue light-activated systems

Historically, optogenetics started with blue and green light because these wavelengths have been studied extensively in relation to photosynthesis in plants. Initially, modified or unmodified opsins found in photoreceptor cells of the retina, light-sensitive bacteria, or algae were used as the light sensors. Studies were also performed on a mammalian opsin, known as melanopsin. This protein is involved in circadian rhythms and the pupillary reflex as well as in retrograde signaling modifying visual function locally within the retina [26, 27]. Melanopsins, along with microbial channelrhodopsins (ChRs), are commonly used optogenetic systems activated by blue light (~ 470 nm) [28]. Below, we will discuss three systems based on blue light activation.

### Melanopsin and NFAT-reporter system

**Fig. 2:**
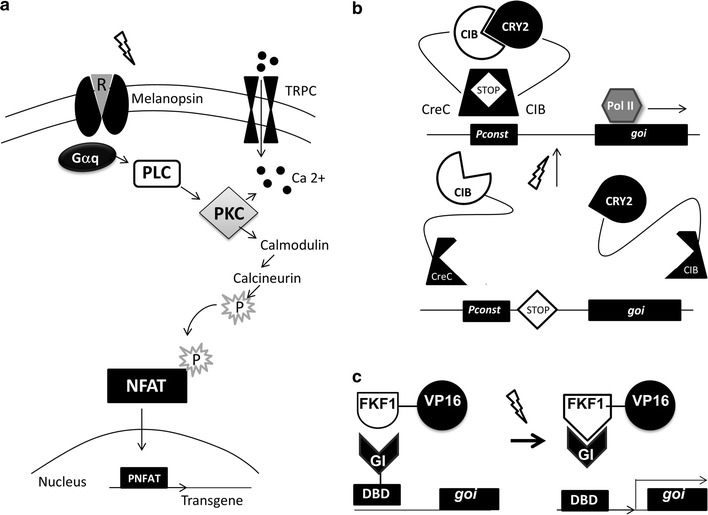
Blue light activated systems. **a** NFAT/Melanopsin system utilizes blue light. The cascade of signaling events opens TRPC to allow calcium ion influx, which, in turn, activates calcineurin that dephosphorylates NFAT and allows for its translocation into the nucleus and the expression of the transgene. **b** CIB/CRY2 systems are activated by blue light and deactivated by darkness. The figure shows how CRY2/CIB1 was used to induce activation of split Cre recombinase by reconstituting the enzyme through its dimerization. Cre is a site-specific recombinase that catalyzes the recombination between two LoxP to excise sequence between them. Cre enzyme was split into two parts: the N-terminal fragment of Cre fused to CRY2 and the C-terminal fragment of Cre fused to CIB1. Blue-light induced interaction between CRY2 and CIB1 lead to the reconstitution of Cre, which then catalyzes recombination at loxP sites. **c** In the absence of blue light, the split transcription FKF/GI factor Gal4 is inactive. In the presence of blue light, VP16 links an active domain to the binding domain region of a transcription factor Gal4 that regains its function and enables expression of the gene of interest. *TRPC* transient receptor protein channels; *Gaq* Gaq-type G protein; *PLC* phospholipase C; *PKC* protein kinase C; *NFAT* nuclear factor of activated T-cells; *CRY2* Cryptochrome 2; *CIB* CRY2-interacting bHLH; *CreC*; *Pol II* polymerase II; *goi* gene of interest; *FKF1* Flavin-binding Kelch repeat F-box; *VP16* activation domain of transcription factor VP16; *GI* GIGANTEA protein; *DBD* DNA binding domain

Melanopsin is a photosensitive molecule that belongs to a subgroup of G-protein-coupled receptors linked to a retinal chromophore. Upon sustained blue light illumination, it converts to metamelanopsin and forms a bistable photosteady state mixture. The absorption maximum of melanopsin is at 467 nm in this mixture, while metamelanopsin has an absorption maximum at 476 nm [28]. Upon intense blue light illumination (λmax = 470 nm), calcium is pumped into the cell from the extracellular space through transient receptor protein channels (TRPCs). The calmodulin reacts to the change in calcium concentration and initiates a signaling cascade [29], where calcineurin dephosphorylates the nuclear factor of activated T-cells (NFAT), thus allowing it to translocate into the nucleus where it binds to the NFAT-responsive promoters (Fig. 2a).

The proof of principle for NFAT-controlled light-inducible transgene expression was demonstrated in HEK-293 cells either cultured in vitro or implanted into mice [29]. The expression of human placental secreted alkaline phosphatase (SEAP) was observed in this system after 3 h of illumination with blue light and maintained with subsequent 3-h light pulses every 24 h. The system reverses to the original state in the dark.

### CRY2/CIB1 system

Cryptochromes represent another class of photoreceptors sensitive to blue light. Structurally, cryptochromes consist of a N-terminal photolyase homology domain and a divergent C-terminal domain. Cryptochrome 2 (CRY2), isolated from *Arabidopsis*, is optimally activated by 350–475 nm light. CRY2 interacts with CRY2-interacting bHLH (CIB1) transcription factor in plant cells illuminated with blue light [30–32]. Experiments with CRY2-mCherry fusions indicate that activated CRY2 interacts with the truncated version of CIB1 fused to a prenylated version of enhanced GFP and relocates to the plasma membrane [30]. In the absence of light, CRY2 and CIB1 do not interact; the CRY2-mCherry complex remains in the cytosol. The system is readily reversible within 10–12 min. Previously recruited CRY2-mCherry fusion protein dissociates from CIB1, returning to its unstimulated state.

The CRY2/CIB1 system has been evaluated both in mammalian HEK-293T cells and in yeast. In mammalian cells, exposure to blue light was employed to induce activation of split Cre recombinase by reconstituting the enzyme through its dimerization [30]. Cre is a site-specific recombinase that catalyzes the recombination between two 34 bp sequences called LoxP to excise sequence between them. In this work the Cre enzyme was split into two parts: the N-terminal fragment of Cre fused to CRY2 and the C-terminal fragment of Cre fused to CIB1. Blue-light induced interaction between CRY2 and CIB1 lead to the reconstitution of Cre, which then performs the recombination at LoxP sites (Fig. 2b). The high effectiveness of this system has been demonstrated in an experiment where expression of a reporter gene EGFP increased 158-fold after 24 h of light exposure [30].

In yeast, the system was designed to induce reassociation of the split Gal4 transcription factor, where the DNA binding domain was fused with CRY2 and the activation domain coupled to CIB1 [5, 30]. When CRY2 and CIB1 were allowed to dimerize after blue light pulses (10 s every 8 min for 4 h), the reconstitution of the Gal4 transcription factor allowed its binding to corresponding DNA target sequences, leading to an a strong increase in expression of a reporter gene [33]. For tissues where blue light does not penetrate efficiently, two-photon approach can be used. This method uses two separate photon beams with lower energy (820–980 nm) which focus on a target and in combination can excite molecules sensitive to blue light. Longer wavelength allows deeper tissue penetration, but requires more energy to reach excitation levels. Employment of two-photon method for CRY2-CIB1 activation approach in rat organotypic hippocampal slices was demonstrated in [30]. In vivo application of the photo-activatable split Cre has been shown in mice in hippocampus, illuminating via optical fibers, and in cortex, via thinned cranial window [34]. This system continues to improve to achieve more robust response to a single light pulse [35] and to reduce baseline activity in the dark [36].

Another two-hybrid system integrating the customizable DNA-binding transcription activator-like effector (TALE) domains from *Xanthomonas sp* with CRY2/CIB1 was developed for the mammalian cells [5, 33]. In a nutshell, light-inducible transcriptional effectors (LITEs) are capable of reversible activation of endogenous genomic loci, while not requiring an exogenous cofactor. This system was tested both in primary mouse neurons and in the mouse brain [5]. Combination of Cry2/CIB1 system with bacterial DNA-binding protein LexA was used to demonstrate light-inducible transcription activation in Drosophila melanogaster—both in cell culture and in vivo [37]. Recently, CRY2/CIB1 activation by blue light was joined with CRISPR technology to create a light-activated CRISPR/Cas9 effector (LACE) system. LACE was successfully employed to simultaneously and reversibly activate human IL1RN, HBG1/2, or ASCL1 promoters in HEK293T cells [33].

### LOV domain systems

A study o*f Arabidopsis thaliana* yielded two more interacting proteins, Flavin-binding Kelch repeat F-box (FKF1) and GIGANTEA (GI) [38]. FKF1 detects light by the binding of the flavin mononucleotide (FMN) to the “light, oxygen or voltage” (LOV) domain. The ground state of the FMN in the LOV domains shows typical absorption spectra of flavoproteins with an absorption maximum from 445 to 450 nm. The activation of LOV can be assumed to cut off effectively above 500–510 nm. Blue light illumination induces formation of a covalent bond between FMN and cysteine 91 on FKF1, allowing its coupling with GI in the nucleus where it can promote transcription of a specially engineered transgene [38]. For example, the DNA binding domain (DBD) of Gal4 may be coupled with GI while FKF1 may be fused to a transcriptional activation (TA) domain of herpes simplex virus protein (VP16) [23]. DBD(Gal4) lacks a dimerization domain and thus is unable to bind its cognate DNA sequence in absence of a TA domain. The GI-DBD(Gal4) would activate the Gal4-binding elements upstream of the transgene after light-induced coupling of DBD(Gal4) and TA domains (Fig. 2c).

The mechanistic aspects of the FKF1-GI system are similar to that of CRY2-CIB1, it is based on coupling of its components after irradiation with blue light [23, 38]. The system takes 30 min to reach the maximum membrane translocation, and is not easily reversible as the dimerization state is maintained for more than 1.5 h after light stimulus is removed. The FKF1-GI system has been used for light-inducible transcription using engineered zinc finger proteins (LITEZ) in mammalian cells [39].

Another example of an LOV-based system allowing manipulation of gene expression is based on the small blue light-sensitive protein vivid (VVD). VVD is fused with both DBD (Gal4) and the transactivation domain of the transcription factor p65 in this system to generate a synthetic protein, termed GAVPO, that can be induced by blue light to homodimerize and, as a dimer, to initiate transcription of genes containing Gal4-binding sites in their promoter region. This transgene system was named LightOn [40, 41]. It could be activated by both continuous illumination and repeated short light pulses. The half-life of the activated GAVPO is approximately 2 h, allowing the temporal control of transgene expression. This system was tested in both model and therapeutic transgene experiments in mice, including LightOn-controlled insulin expression [41].

In a recent elegant work, LOV2 domains of *Avena sativa* phototropin 1 (*As*LOV2) were fused with two different protein domains that impair functioning of the repressor element 1 (RE1)-silencing transcription factor (REST), a master regulator of neural genes. Both chimeras specifically inhibited REST activity on illumination, causing an increase in the transcription of REST target genes and enhanced firing activity in primary neurons [42].

It is important to note that LOV domain containing proteins are encoded in genomes of plants and algae as well as some fungi and bacteria. Optimization of LOV-based systems may clearly benefit from bioprospecting efforts. An engineered bacterial DNA-binding LOV protein EL222 resulted in a luciferase reporter systems with highly linear response to blue light, rapid activation (< 10 s) and deactivation (< 50 s) kinetics and a large (> 100-fold) dynamic range of target protein expression in mammalian cells and zebrafish embryos [43].

## Green light activated systems

A green-light based system requires additional manipulation, similarly to blue light, to make it clinically applicable, due to poor green light penetration into tissues. Hence, a typical application of a green-light inducible system is for the activation of bioprocessing organisms, such as algae or bacteria, to produce biofuels or biomaterials.

### CcaS/CcaR system

**Fig. 3:**
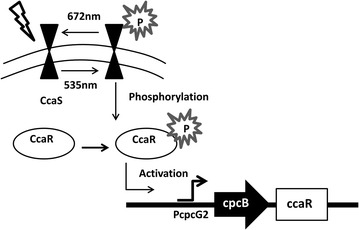
Green light inducible system. In this example of the green light inducible system, CcaS becomes phosphorylated upon illumination at 535 nm as green light activates the system. However, upon red light stimulation the cognate response regulator CcaR is dephosphorylated and the transgene expression is switched off. *CcaS* Cyanobacteriochrome, *CcaR* downstream cyanobacteriochrome regulator; *pcpcG2* promoter of gene cpcG2 that is regulated by CcaR; *cpcB* 5′UTR sequence derived from the cpcB gene; *ccaR* ccaR gene

The CcaS/CcaR system utilizes a green-light responsive photosensing histidine kinase CcaS from cyanobacteria *Synechocystis* and its cognate downstream regulator CcaR. The CcaS/CcaR system is used to induce the expression of a phycobilisome linker gene (cpcG2) in cyanobacteria [44, 45]. This system’s activation and deactivation are controlled by two different wavelengths of light: green light (535 nm) activates the system, but red light (672 nm) stimulation causes the cognate response regulator CcaR to be dephosphorylated [44], thereby switching off transgene expression (Fig. 3). Interestingly, the native promoter of the cpcG2 gene was not induced very efficiently, so the enhanced promoter was engineered to bring expression levels up 40-fold with a high ON/OFF ratio [44].

Similarly designed photoswitchable systems that use green/red light switches were built by construction of chimeras. For example, cyanobacterial phytochrome Cph1 and the genes of the EnvZ/OmpR signaling pathway were combined in *E. coli* to express a phycobilisome-related gene [45]. Coupling the CcaS/CcaR to a red phytochrome sensor Cph8 allows for multichromatic transgene expression, because CcaS is inactivated in the red band to which the Cph1/EnvZ chimera Cph8 responds. Hence, green and red light could be differentially applied to specifically induce transcription in batch culture or in patterns across a lawn of engineered cells [45, 46].

## Red/NIR light activated systems

Although green and blue light activated optogenetic constructs work well in cell culture and transparent animals, their use in heme-rich animal tissues is problematic due to light scattering and absorbance. Red and near-infrared (NIR) light-responsive systems are better for clinically relevant applications, due to the fact that NIR light efficiently penetrates tissues to depths of several centimeters as compared to visible and ultraviolet wavelengths [21, 22]. High tissue penetration and almost nonexistent toxicity makes NIR an optimal light range to regulate gene transcription for therapeutic purposes. It should be also noted that the skull and the brain are much more transparent to red and NIR light than hemoglobin-rich peripheral tissues [21]. The depth of penetration of the brain tissue further increases to the depth more than 5 cm when a red light-emitting diode (LED) array is wrapped over the crown to benefit from the convergence of light from adjacent LED elements [47, 48].

### BphG1/DGCL system

NIR-responsive systems are often based on engineered bacterial phytochromes (BphPs), whose maximal absorption wavelengths are red shifted as compared to those of plant and cyanobacterial phytochromes. Thus, use of BphPs enhances the responsiveness of optogenetic sensors deep within mammalian tissues [49].

**Fig. 4:**
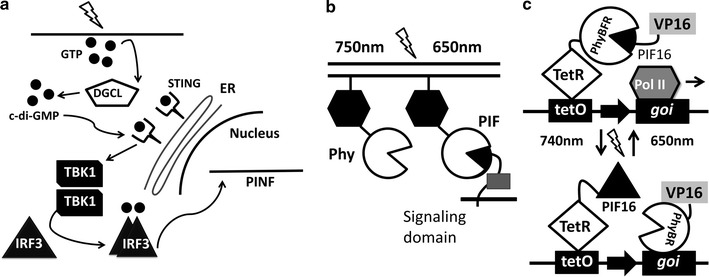
Red light-activated systems. **a** Near-infrared light is detected by BphG1 which triggers the release of GMP and secondary messenger c-di-GMP. The c-di-GMP is recognized by STING and activates the phosphorylation of IRF3 by TBK1. IRF3 then translocates into the nucleus, binds to IRF3-specific operators and induces the IFN controlled promoters. **b** PhyB/PIF system is activated by 650 nm and deactivated by 750 nm. In this split system, PhyB component is bound to the membrane, while PIF3 is in the cytoplasm. Light activation results in the dimerization of PhyB/PIF and the translocation of PIF-bound protein of interest to the nucleus. **c** The system incorporates VP16 and the Tet operon with PhyB and PIF. In the TetR-PIF6 split transcription factor construct, the N-terminal fragment of nuclear-targeted PhyB is fused to the VP16 TA domain, and the N-terminal of PIF6—to the tetracycline repressor TetR. To control expression of the gene of interest, the TetR-specific operator, TetO, is inserted into its promoter. Illumination with 650 nm light leads to the reversible heterodimerization of PhyB with PIF6. Since PhyB is fused to TetR, heterodimerization brings VP16 in vicinity of TetO, initiating transcription of the gene of interest. After far-red (740 nm) light-induced conversion of PhyB into its inactive form, the dissociation of PhyB-PIF6 inhibits expression of the transgene. *BphG1* Rhodobacter sphaeroides phytochrome BphG1; *GMP* Guanosine monophosphate; *GTP* Guanosine triphosphate; *c*-*di*-*GMP* cyclic diguanylate monophosphate; *STING* stimulator of interferon genes; *IRF3* interferon regulatory factor 3; *TBK1* tank-binding kinase 1; *DGCL* diguanylate cyclase; *ER* endoplasmic reticulum; *PINF*(*ACD *+) interferon promoter; *Phy* phytochrome; *PIF* phytochrome-interacting factor; *TetO* tetracycline operator; *tetR* tetracycline repressor; *VP16* activation domain of transcription factor VP16; *PIF16* phytochrome interacting factor 16; *PhyBFR* active FR form of phytochrome B; *Pol II* polymerase II; *goi* gene of interest

Truncated phosphodiesterase (PDE) domain from *Rs*BphG1 associates with diguanylate cyclase (DGCL) and empowers it to convert GTP to cyclic diguanylate monophosphate (c-di-GMP), the secondary messenger molecule. In turn, c-di-GMP triggers activation of stimulator of interferon genes (STING), thus enabling phosphorylation of interferon regulatory factor 3 (IF3) followed by its translocation into the nucleus where it binds to interferon-β regulated promoters [50] (Fig. 4a). This system has been recently used in an elegant work on an electroencephalography-based brain–computer interface in mice capable of processing mental state-specific brainwave programs. It was inductively linked to a wireless-powered optogenetic implant containing cells engineered for NIR light-adjustable expression of SEAP transgene [50].

Another photosensory bacteriophytochrome, BphP1 from *Rhodopseudomonas palustris,* has NIR spectra and the highest fluorescence quantum yield (ratio of the number of photons emitted to the number of photons absorbed which is proportional to fluorescence output, “brightness”) among other BphP-derived light sensors [51]. Upon NIR illumination, *Rp*BphP1 heterodimerizes with the transcriptional repressor RpPpsR2, an interaction that requires biliverdin IXα chromophore endogenously synthesized in many mammalian cells. This system was utilized to induce cellular cytoskeletal rearrangements by the recruitment of a *Rp*BphP1-linked Dbl homology (DH) and Pleckstrin homology (PH) domains of intersectin 1 to the plasma membrane with subsequent activation of the small GTPase Cdc42, and to design light-inducible tetracycline repressor/operator constructs driving expression of SEAP [51]. *Rp*BphP1/*Rp*PpsR2-based systems minimally interfere with the metabolism of mammalian cells, which make them very well-suited for therapeutic applications. Further development of this system was undertaken to find minimal 17 kDa functional domain of *Rp*PpsR2 and improve signal to noise ratio in mammalian cells [52].

### PhyB/PIF system

The plant phytochrome PhyB with a photosensitive N-terminal domain was cloned from *Arabidopsis thaliana*. In an optogenetic split transcription factor system, a fragment of PhyB is conjugated to the membrane permeating chromophore phycocyanobilin (PCB). Under exposure to 650 nm light, PhyB conjugate dimerizes with PIF3, a helix–loop-helix protein [53] and translocates to the nucleus to affect downstream targets [54] (Fig. 4b). Exposure to far-red light (750 nm) induces dissociation of PIF3/PhyB dimers and, therefore, dissociation of binding and activation domains, rendering the transcription factor assembly non-functional.

PhyB/PIF systems have been implemented for both yeast [20, 54] and mammalian cells [53, 55]. Red light induced two-hybrid activation of Gal4 in yeast cells could be fine-tuned by titrating the number of photons delivered to the cells by the pulsation of light. The system is photoreversible, giving it an advantage over systems with slower reverse kinetics [54]. The detection of red light is dependent on a chromophore, PhyB, which is not present in yeast or animal cells. Therefore an analogous bilin, phycocyanobilin, extracted from the cyanobacterium *Spirulina*, is supplemented to culture media as a substitute for phytochromobilin [55]. The required addition of a bilin ligand is a limitation of the PhyB-PIF system in vivo.

### TetR-PIF6 system

Activation of transcription based on tetracycline (Tet)-dependent promoter constructs has been used in biological research for a long time. In Tet-systems, induction and deactivation of expression are dependent on either the addition or removal of tetracycline or its analogue doxycycline [56]. The performance of these systems is inferior to that of their light-controlled analogues due to the poor temporal control and substantial chemical diffusion time. A typical Tet-OFF system uses a TetR-DNA binding protein and a VP16 TA domain from *Herpes simplex* virus fused to its operator site [56, 57]. Other derivatives of VP16, like VP64 which possesses a tetrameric repeat of the minimal VP16 motif, are sometimes employed. The VP16 motif recruits subunit 25 of the Mediator complex (Med25). Overexpression of the Med25-VP16 binding domain (Med25VBD) competitively inhibits VP16, and subsequently suppresses the transgene [58].

The limited clinical applicability of blue light required modification of the Tet-OFF system to make it red-light inducible. In the TetR-PIF6 split transcription factor construct, the N-terminal fragment of nuclear-targeted PhyB is fused to the VP16 transactivation domain, and the N-terminal of PIF6 is fused to the tetracycline repressor TetR [55, 58]. The TetR-specific operator, TetO, should be inserted into transgene’s promoter to control expression of the transgene of interest (Fig. 4c). As mentioned above, the PhyB-PIF6 system is activated when red light (660 nm) converts PhyB (amino acids 1–650) into its active FR form (PhyBFR) and reconstitutes a split transcription factor. This results in the reversible heterodimerization of PhyB with PIF6 fused to TetR, and, consequently, to the VP16 initiation of the transcription in vicinity of TetO. After far-red (740 nm) light-induced conversion of PhyB into its inactive form, the dissociation of PhyB-PIF6 inhibits expression of the transgene. TetR is not induced by VP16 in absence of the dimerization of PhyB and PIF6 and the target promoter is not activated, resulting in silencing of the transgene.

This system has been applied to guide gene expression in various mammalian cell lines [55]. Low intensity pulses of light (80 nmol cm^−2^ total photon count) were sufficient to switch gene expression on and off [55]. Importantly, experiments with human umbilical vein endothelial cells (HUVECs) showed that the expression levels of the target gene are proportional to chromophore concentration and the number of absorbed photons. In one study, Chinese hamster ovarian cells (CHO-K) were transiently transfected with the light inducible split transcription factor and a human vascular endothelial growth factor splice variant 121 (hVEGF121) reporter, then illuminated with 660 nm (red) or 740 nm (far-red) light for 24 h. The expression of a transgene of interest, hVEGF121, was observably modulated [55].

The TetR-PIF6 system was later used to control hVEGF121 production in vivo [55]. To achieve that, the transfected CHO-K1 cells were embedded into a biocompatible polyethylene glycol (PEG)-based hydrogel and applied onto the chorioallantoic membrane of 9-day old chicken embryos. The embryos were stimulated for 48 h with red or far-red light. At the end of the 48 h period, the embryos which were exposed to 660 nm illumination displayed phenotypes of hVEGF121-induced neovascularization and angiogenesis [55]. The embryos that were stimulated with far-red light did not display any markers of angiogenesis, consistent with the absence of target gene expression. The red-light-inducible gene expression system can be optimized by increasing the copy number of the TetO sequence in the reporter construct and increasing the distance of the TetO from the minimal promoter [55]. The photoreversibility of the system at 740 nm is fast and comparable to the turnaround of tetracycline-induced termination of transcription through the dissociation of TetR from TetO. However, the activation of PhyB by red light is sustained in the dark, and its reversion follows a slower kinetic that does not have significant impact on shutting off transgene expression. Reversibility, adjustability, and temporal and spatial control make the TetR-PIF6 system an attractive candidate for biomedical applications.

## Potential therapeutic applications of optogenetics

Recently, a number of technological developments in optogenetics paved the way for chemical-free manipulation of transgene expression in mammalian cells. At first, optogenetic systems aimed to fill a niche as the method of choice for studying the pathophysiology of neurological and neuropsychiatric disorders in a range of animal models [7]. Gene therapy field started to develop optogenetic approaches for medical conditions such as pharmacoresistant epilepsy [10] and inherited retinal degeneration [11]. High tissue penetration of the red and NIR light makes optogenetic tools attractive for the knee joint, smaller joints and the adult brain, since light can be efficiently delivered by a LED array wrapped around the knee [59] and smaller joints or in a helmet-type configuration [13] applied to the head.

The advantage of light over chemical regulators (such as tetracycline) is that light-based treatment can be focused on a particular small area, is easy to deliver, and allows for the control of dosage and timing [60]. In therapy, OPT could be eventually employed to control localization, timing, and dosage in the expression of a therapeutic transgene. Another potential application could be the creation of grafts that produce a secretable factor under the control of light. A co-expression of all necessary transgenes in engineered stem cells that are then used as the therapeutic vehicle is an emerging approach [61]. An engineered stem cell would carry the entire expression system, and upon activation by light, would transcribe and secrete therapeutic proteins to be released near their sites of action, allowing for an external control of expression of therapeutic proteins using repeated light activation.

## Limitations to clinical translation of optogenetics-based systems

There are considerations that are important to address in the utilization of light rather than a chemical to induce transgene expression. Aside from the general concerns of cell type, excitation wavelength, duration of activation, and photo-reversibility of the system, other components of the synthetic circuit machinery may also impact the efficacy of gene expression.

### Properties of photosensory domains

The large size of some phytochrome or cryptochrome photosensory domains is hard to accommodate into viral vectors. For example, the PHR domain of CRY2 used in the CRY-CIB system is 498 amino acids and PhyB, part of the PhyB-PIF6 system, is 1172 amino acids [62, 63]. Recently the reduced version of RpBphP1/RpPpsR2 system, RpBphP1/Q-PAS1 has been developed, where the enhanced RpBphP1/Q-PAS1 transcription activation construct was shown to be 2.5 kbp smaller than the original RpBphP1/RpPpsR2 system [52]. Conformation of the protein domain may also dictate whether it would be working as a fusion. For example, PhyB will only heterodimerize at the C-terminus, while CRY2 preferentially binds to CIB only when its conformation is intact, hence limiting possible fusion constructs. Some proteins form constitutively active dimers and that makes them unsuitable candidates for optogenetics. Smaller heterodimerizing photosensory protein fragments such as LOV domains are better candidates for incorporation into viral vectors. On the other hand, developing more capacious vectors can also be instrumental in overcoming this challenge.

### Photo-responsive wavelength

The choice of wavelength for therapeutic applications is guided by several considerations. The excitation wavelength should not be toxic to the cells on its path. High frequency electromagnetic waves (e.g. UV light) have a short wavelength and high energy, while low frequency waves (e.g. NIR) have long wavelengths and low energy. UV light can cause DNA damage; at high exposure blue light is also cytotoxic, in particular, blue light-dependent induction of intracellular oxidative stress could contribute to premature skin photoaging [64]. Depth of penetration is also important for high resolution and precise spatio-temporal control; red light and far-red light have greater penetrance than blue and green light, thereby making systems like PhyB-PIF6 clinically relevant. Of interest are recent publications on red-shifted channelrhodopsins [65, 66] and Jaws, a red light sensitive opsin from *Haloarcula salinarum* (strain Shark) [67]. The latter has already been utilized to restore photosensory retinal responses with greater spike rates and in a naturalistic frequency range [67]. Another concern is that the reporter protein often transmits a wavelength which overlaps with the excitation wavelength. For example, if the transgene encodes a fluorescent protein such as GFP, the cross reactivity of the two light-regulated proteins may disrupt the control of transgene expression. Multiple switches can be engineered into the system by using photoactive proteins responsive to different excitation wavelengths. The use of a different wavelength in red/green light or red/far red systems, can serve as an ON/OFF trigger that makes activation and deactivation of the system substantially faster.

### Target cells

Many optogenetic studies use bacterial model systems instead of mammalian cells because the secondary orthogonal messenger required for signal transduction is not endogenously produced by certain cell types. Photoreceptors dependent on an exogenously added chromophore may have limited potential for seamless integration into the host’s signaling cascades. In this sense, *Rp*BphP1-based sensors are of high interest as they rely on mammalian biliverdin IXα chromophore [51, 53]. Engineering of stably transfected mammalian cells for later implantation is relevant to future clinical applications, but also a challenge because viral vectors may require specific surface receptors for entry [68] and can be immunogenic [68] or cause insertional mutations [69].

## Conclusions

Optogenetic approaches offer an opportunity for temporal, dosage and spatial control. Light-controlled expression of various transgenes gained substantial traction in research labs and aided in gaining insights into physiology and pathophysiology in the past decade. As more genomes are sequenced through bioprospecting, more light-induced protein interactions will be found and used as optogenetics building blocks. The optogenetic system should be independent of exogenous chromophores, able to accommodate larger transgenes, and capable of light-dependent activation in deeper tissues to suit a number of applications. The engineering of designer cells for subsequent transplanting into the body is a promising way for delivery of optogenetic constructs. Importantly, light-dependent actuators may be combined with existing technologies; for example, the detectors for EEG brain waves, ion potentials, or levels of certain neurotransmitters could be incorporated into a biosynthetic circuit where light is produced proportionally to the signal and is harnessed to induce the expression of a therapeutic transgene that may be regulated by a feedback loop. Primary clinical areas for adoption of optogenetics treatments are disorders of the brain, including those characterized by seizures or other intermittent symptoms, cardiac arrhythmia, and diabetes that requires on-demand control of blood glucose. The optogenetic paradigm is especially attractive as it steps away from predominantly pill-based approaches towards much more targeted therapy by providing control over timing, location and delivered dosage of biologic drugs.
